# Nitrate in Polluted Mountainous Catchments with Mediterranean Climates

**DOI:** 10.1100/tsw.2001.324

**Published:** 2001-12-07

**Authors:** Thomas Meixner, Mark Fenn, Mark Poth

**Affiliations:** Department of Environmental Sciences, Unversity of California, Riverside 92521, USA

**Keywords:** catchment, forested ecosystems, hydrology, nitrogen deposition, Mediterranean climate, semiarid

## Abstract

The mountains of southern California receive some of the highest rates of nitrogen (N) deposition in the world (~40 kg ha year). These high rates of deposition have translated into consistently high levels of nitrate (NO3) in some streams of the San Bernardino Mountains. However, not all streams are exhibiting these high levels of NO3. Perennial streams have high NO3 concentrations (~200 μmoles l) while ephemeral streams do not (~20 μmoles l). This difference points to groundwater as the source of the NO3 observed in streams. Furthermore, the evidence indicates a differential impact of N deposition on terrestrial and aquatic systems in Mediterranean climates, with aquatic systems being impacted more quickly. The primary reason for this difference involves the asynchrony between the time that atmospheric deposition occurs (summer), the time period of maximum soil NO3 availability and leaching (winter), and the time of maximum plant N demand (spring). Our results indicate that semiarid Mediterranean climate systems behave differently from more humid systems in that, because of this asynchrony, aquatic systems may not be indicative of changes in terrestrial ecosystem response. These differences lead us to the conclusion that the extrapolation of impacts from humid to Mediterranean climates is problematic and the concept of N saturation may need to be revisited for semiarid and seasonally dry systems.

## INTRODUCTION

The study of the ecosystem effects of atmospheric nitrogen (N) deposition and its effect on terrestrial aquatic ecosystems has received a great deal of attention in recent years[1,2,3,4]. These studies have generally identified high levels of nitrate ($NO_3^-$) observed in stream baseflow in the summer as indicative of the latter stages of N saturation (stage 2). The N saturation hypothesis as propounded by Stoddard[3] thus argues that stream chemical composition, particularly $NO_3^-$ concentrations, is indicative of terrestrial ecosystem processes. This idea underlies much of watershed biogeochemistry[5]. However, much of the study on the effects of N deposition (and more broadly watershed biogeochemistry) have focused on relatively humid systems with a few notable exceptions[6,7,8]. This lack of consistent work on N concentrations and fluxes in seasonally dry and semiarid systems has not permitted a robust verification of the N-saturation hypothesis outside of the more mesic climates where the hypothesis originated.

Previous work in southern California indicates that normal $NO_3^-$ concentrations in the southern California streams should be around 5 or 10 μmoles l$^{-1}$ with a small seasonal pulse of N in some watersheds[4]. We might therefore consider values above

30 µmoles $l^{-1}$ abnormally high for streams in southern California. In the Devil Canyon catchment in the western San Bernardino Mountains, 100 km east of Los Angeles, streamwater $NO_3^-$ concentrations have been monitored since late 1995. This watershed receives some of the highest rates of N deposition in the world (~40 kg $ha^{-1}$ $year^{-1}$). Although N deposition is similar throughout the Devil Canyon watershed, $NO_3^-$ concentrations vary considerably among the sampling sites. This variability provides a unique opportunity to determine the biogeochemical and hydrologic factors that exert the greatest control on $NO_3^-$ concentrations from Mediterranean forested catchments with elevated N deposition. In addition to N concentrations, $Ca^{2+}$ and dissolved organic carbon (DOC) concentrations were monitored, since they have shown previous success at identifying how biogeochemical processes affect streamflow[9,10]. In this paper we seek to answer two questions regarding $NO_3^-$ concentrations in seasonally dry catchments:

1. What can the variability in $NO_3^-$ concentrations at Devil Canyon tell us about the processes controlling inorganic N in seasonally dry watersheds?
2. What implications do these results have for the concept of applying the N saturation concept to seasonally dry systems?

## METHODS AND PROCEDURES

### Site

Streamwater samples were collected for chemical analysis from eight streamwater sampling sites in Devil Canyon, a catchment heavily impacted by atmospheric deposition from the urban Los Angeles air mass (Fig. 1). These sites had been previously studied by Fenn et al.[4]. Camp Paivika is located at the top of the watershed at 1580 m, while the USGS stream gauge station (Station no. 11063680) is at an elevation of 632 m. Vegetation is mixed conifer near the crest of the watershed but grades to chaparral at lower elevations with riparian areas dominated by white alder (*Alnus rhombifolia Nutt.*); intermediate elevation and riparian zones have large areas of California bay laurel (*Umbellularia californica*), scrub oak (*Quercus dumosa*), and coast live oak (*Q. agrifolia*). The geology of the Devil Canyon watershed is composed of crystalline and sedimentary rocks ranging from ancient Precambrian to quaternary deposits. Much of Devil Canyon is underlain by plutonic igneous rocks of Mesozoic age, mostly quartz monzonite and granodiorite[8].

Estimates of atmospheric deposition of N vary for the Devil Canyon watershed but range between 35 and 45 kg $ha^{-1}$ $year^{-1}$.

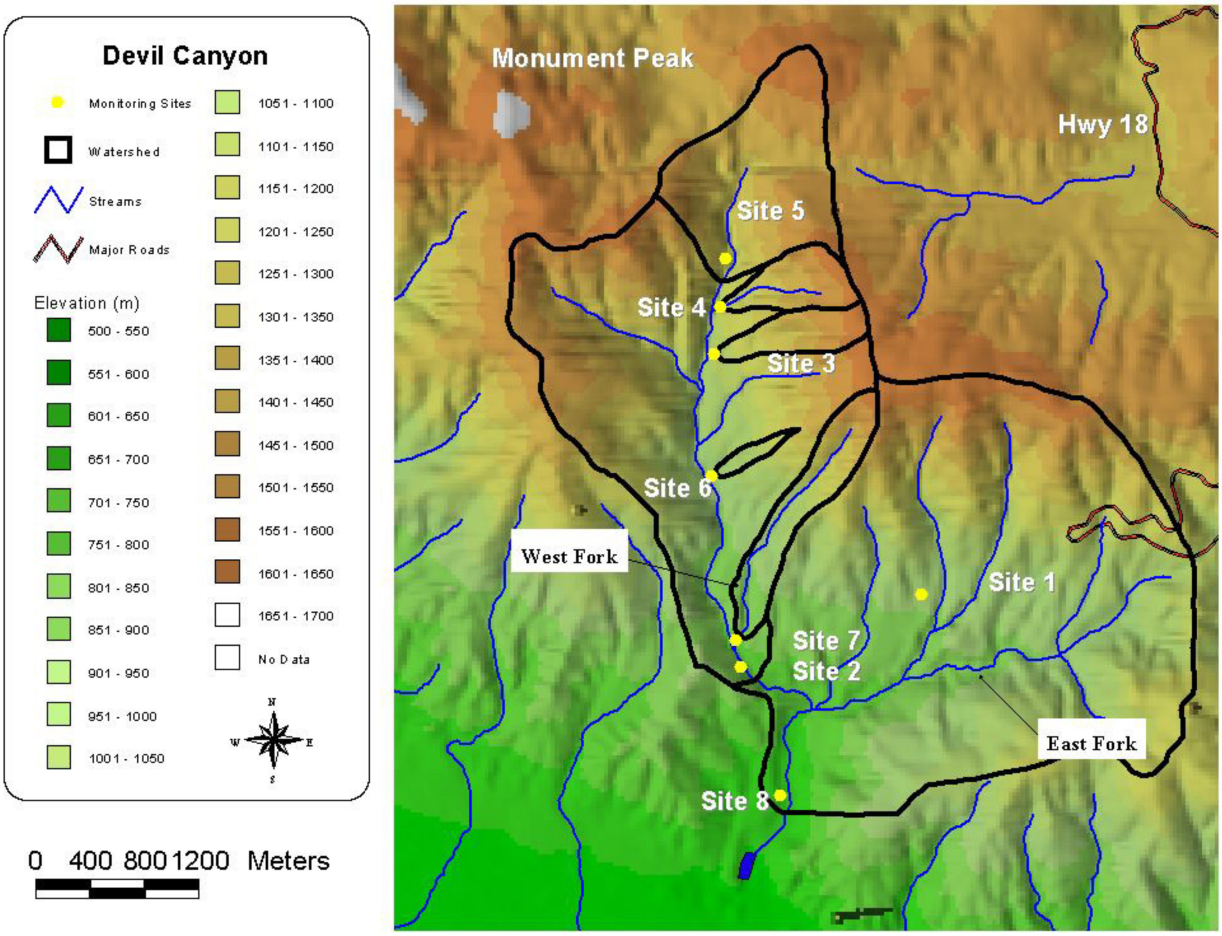

**FIGURE 1.** Site locations in the Devil Canyon watershed. Site 8 is coincident with a USGS gauging station.

Mean annual precipitation for the watershed varies from 610 mm $year^{-1}$ at the bottom of the watershed to 987 mm $year^{-1}$ at the top of the watershed at Camp Paivika. Precipitation is profoundly seasonal, with more than 80% of precipitation occurring as rain during the period from December to March (Fig. 2). The eight sites being sampled vary greatly in watershed area and dominant riparian and watershed-wide vegetation types, as well as the steepness of the watershed (Table 1)[8].

## Field and Laboratory Methods

The eight sites being monitored have been sampled to varying degrees since 1996. Sampling and analysis previous to 2000 was reported in Fenn and Poth[8]. Our analysis follows the procedure outlined below: grab sampling was conducted at all eight sites on an approximately weekly basis in winter and biweekly basis in summer. Simultaneous to all grab sampling, streamflow was measured either by the velocity profile method or by recording the time taken to fill a container of known volume. Grab sampling was supplemented by the use of Sigma MAX 900 autosamplers, which are designed to take samples on a daily basis in general, although some event sampling was also conducted. Daily and event sampling is always present for site 2, while a second autosampler was moved between sites such that we attempted to capture a significant rain event for each of the sites. Grab samples were filtered (0.45 µm) immediately in the field into triple-rinsed plastic bottles. Separate samples were collected for organic carbon analysis. Organic carbon samples were collected in combusted glass bottles with Teflon-lined lids. Organic carbon samples were filtered with combusted glass fiber filters (Whatman GF-F) within 2 days of returning from the field. All

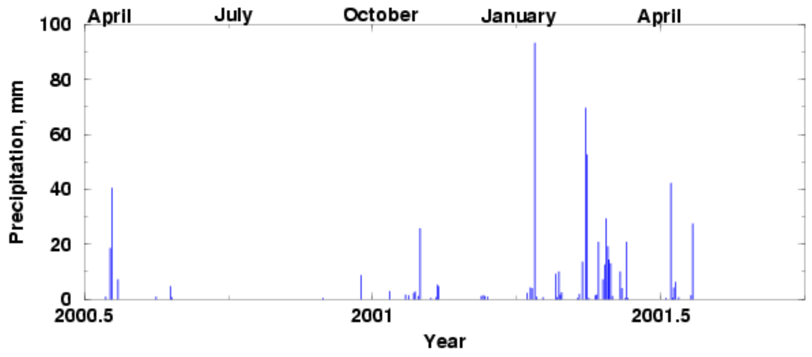

**FIGURE 2.** Daily rainfall at Devil Canyon in 2000 and 2001. Typically 80% of rainfall occurs between December and March.

**TABLE 1**
**Watershed Characteristics**

| Watershed | Area (ha) | Watershed Slope (%) | Conif.[1] Cover (%) | Hard.[2] Cover (%) | Chap[3] Cover (%) | Riparian | Flow Regime |
|---|---|---|---|---|---|---|---|
| Site 1 | N/A | N/A | 5 | 12 | 77 | N/A | Groundwater spring |
| Site 2 | 667 | 49 | 12 | 22 | 65 | Alder | Groundwater dominated |
| Site 3 | 28 | 49 | 27 | 39 | 34 | Ephem.[4] | Ephemeral stream with shallow flowpath |
| Site 4 | 31 | 50 | 36 | 56 | 8 | Alder | Groundwater dominated |
| Site 5 | 137 | 47 | 67 | 21 | 12 | Alder | Groundwater dominated |
| Site 6 | 6 | 51 | 0 | 1 | 99 | Bay | Ephemeral stream with shallow flowpath |
| Site 7 | 52 | 50 | 13 | 4 | 81 | Chap.[3] | Groundwater dominated |
| Site 8 | 1421 | 50 | 10 | 15 | 75 | Sycamore | Groundwater dominated |

[1] Coniferous.
[2] Hardwood.
[3] Chaparral.
[4] Ephemeral.

samples were stored in the dark at 4°C until analysis; DOC and N analyses were completed within 1 month of field collection.

A Technicon TRAACS 800, Autoanalyzer (Tarrytown, NY) was used to analyze for nitrate, ammonium, and phosphate. Sample preparation involved filtering the samples using a 0.45-µm membrane filter. A Varian ICP-OES was used with filtered and acidified samples for the analysis of $Ca^{2+}$ concentrations. Total DOC concentrations were determined by injecting 1 ml of filtered sample into a Dohrmann, which utilizes persulfate oxidation of organic carbon and ultraviolet radiation to determine the amounts of DOC in the samples. NIST standards and blind spikes were used to ensure the accuracy of the analysis.

## RESULTS AND DISCUSSION

Concentrations of $NO_3^-$ vary from almost undetectable to almost 360 µmoles $l^{-1}$ among the eight sites (Figs. 3 and 4). Besides this variability among the sites, there is additional temporal variability, with the highest concentrations observed during winter rains and most particularly with the highest concentrations observed during the highest flows. The sites group into categories that will help further discussion. Sites 1, 3, 4, 6, and 7 are tributaries to the main stem of Devil Canyon, while sites 5, 2, and 8 (in that order) are a downstream transect along the stream (Fig. 1). Both site 1, a spring sampled at the source, and site 7, a relatively constant flow stream with a long course and narrow watershed (Fig. 1), have relatively stable $NO_3^-$ concentrations (average values of 50 µmoles $l^{-1}$ for site 1 and 70 µmoles $l^{-1}$ for site 7) throughout the year (Fig. 3). The three sites with the largest area and largest flow (sites 2, 5, and 8 [USGS site]) have a distinctive rise in concentration in the winter months (as high as 220, 360, and 105 µmoles $l^{-1}$, respectively) and a subsequent decline in concentration during the summer months (to minimum concentrations of 1, 16, and 25 µmoles $l^{-1}$, respectively) (Fig. 4). The two sites with relatively little flow and the lowest concentrations (often below detection limits), site 6 and site 3, are similar in their low levels of inorganic N; however, Site 3 has a notable flushing of $NO_3^-$ (peak concentration of 105 µmoles $l^{-1}$) at the onset of flow (site 3 is in an ephemeral stream), while concentrations at site 6 are remarkably stable (Fig. 3). Site 4 is somewhat of an intermediate stream; it has an annual variation in $NO_3^-$ concentration, but its concentration values (with a peak of 66 µmoles $l^{-1}$ and a minimum of 15 µmoles $l^{-1}$) are smaller than the large sites (2, 5, and 8) and somewhat higher than the two shallow flow-dominated sites, 3 and 6 (Figs. 3 and 4).

Looking at $NO_3^-$ variation vs. flow, as well as $Ca^{2+}$ and DOC concentration vs. flow, helps illustrate the dominant processes governing $NO_3^-$ concentrations in these eight catchments (Table 2). A strong correlation between $NO_3^-$ and DOC concentration and flow is seen at sites 2, 4, 5, and 8 ($r^2$ of 0.85, 0.48, 0.63, and 0.86 for $NO_3^-$; 0.55, 0.45, 0.65, and 0.47, respectively, for DOC). Conversely, there is generally a negative correlation between flow and $Ca^{2+}$ concentration at these same sites

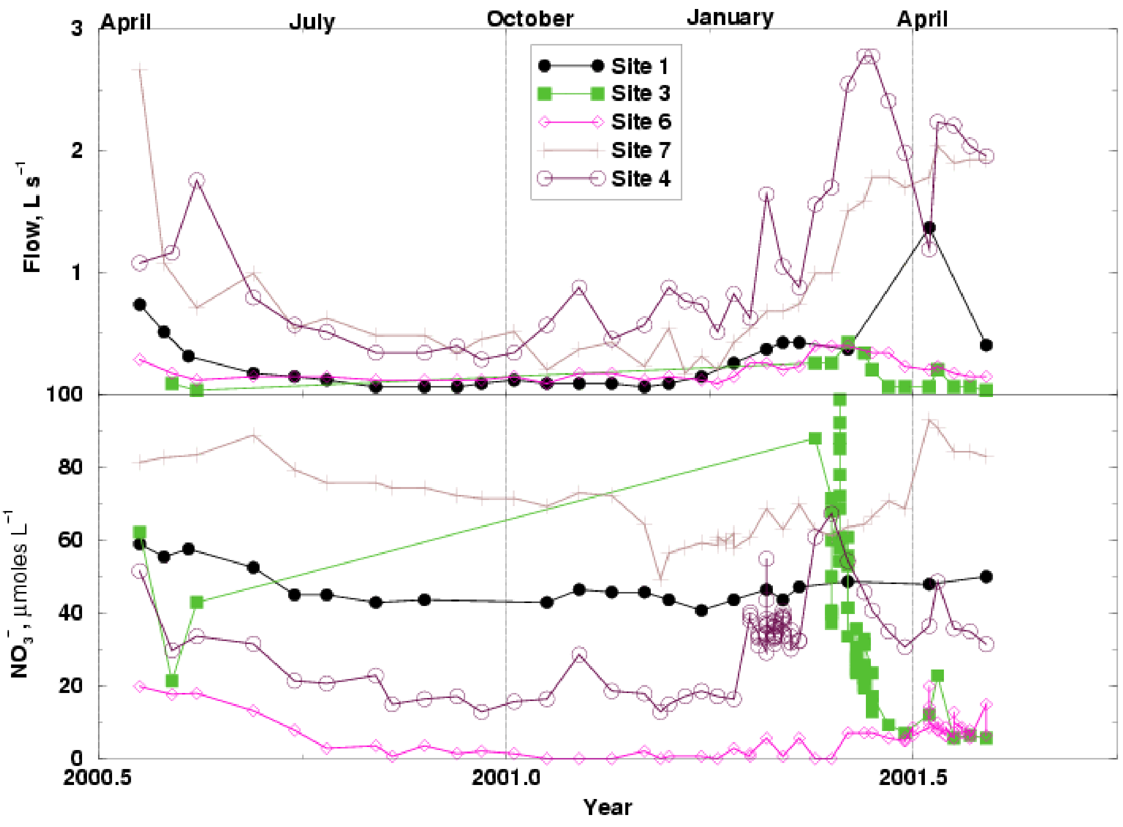

**FIGURE 3.** Stream discharge and $NO_3^-$-N concentrations for sites 1, 3, 4, 6, and 7 in the Devil Canyon watershed. Note relatively stable $NO_3^-$ concentrations for site 1, 6, and 7 and pronounced flushing event for site 3. All temporal data is shown in water years. October 1 represents the beginning of the water year.

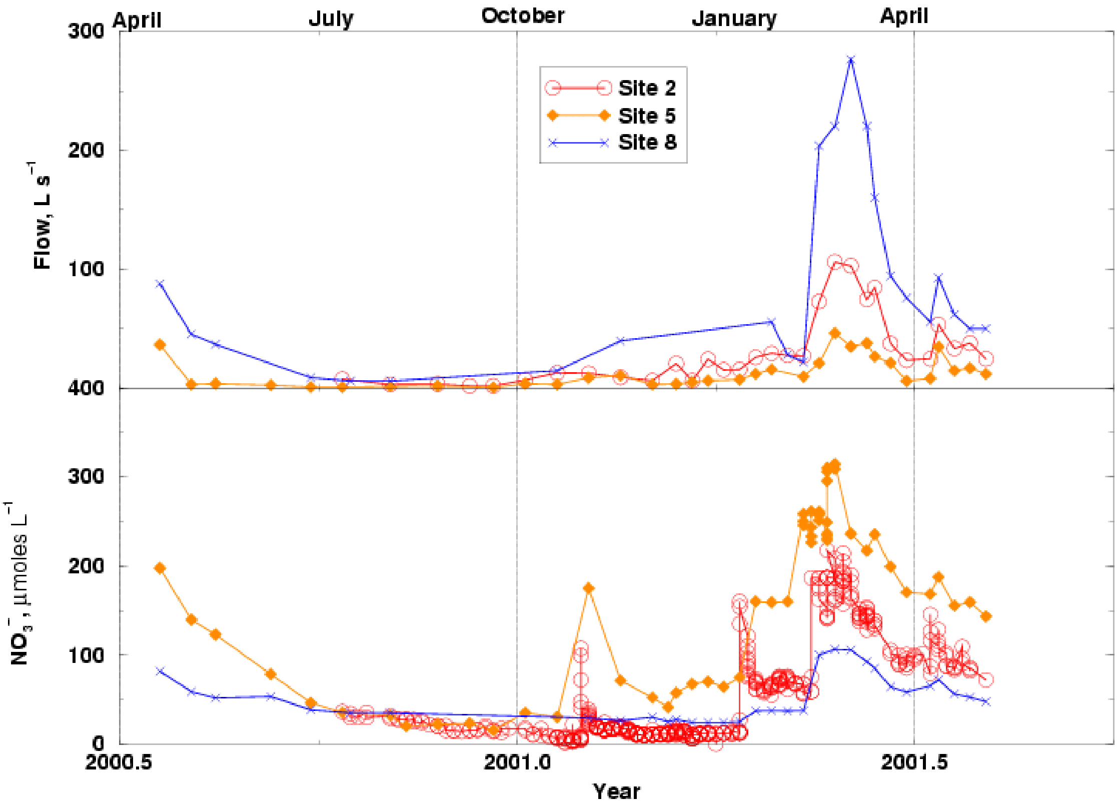

**FIGURE 4:** Stream discharge and $NO_3^-$ as N concentrations for sites 2, 5, and 8 in the Devil Canyon watershed. Note pronounced seasonality of $NO_3^-$ concentrations for all of these sites and correlation of this seasonality with flow.

**TABLE 2**
**Regression Results for DOC, $NO_3^-$, and $Ca^{2+}$ against Stream Discharge**

| | DOC vs. Flow | | | $NO_3^-$ vs. Flow | | | $Ca^{2+}$ vs. Flow | | |
|---|---|---|---|---|---|---|---|---|---|
| Site | Pearson R | $r^2$ | Bonferroni P | Pearson R | $r^2$ | Bonferroni P | Pearson R | $r^2$ | Bonferroni P |
| 1 | 0.363 | 0.132 | 0.097 | 0.677 | 0.458 | 0.001 | –0.118 | 0.014 | 0.598 |
| 2 | 0.745 | 0.555 | 0.000 | 0.926 | 0.857 | 0.000 | –0.464 | 0.215 | 0.017 |
| 3 | 0.691 | 0.477 | 0.009 | 0.557 | 0.310 | 0.038 | –0.501 | 0.251 | 0.140 |
| 4 | 0.671 | 0.450 | 0.000 | 0.689 | 0.475 | 0.000 | –0.298 | 0.089 | 0.097 |
| 5 | 0.804 | 0.646 | 0.000 | 0.797 | 0.635 | 0.000 | –0.837 | 0.700 | 0.000 |
| 6 | 0.451 | 0.203 | 0.008 | 0.045 | 0.002 | 0.791 | –0.228 | 0.052 | 0.226 |
| 7 | 0.545 | 0.297 | 0.001 | 0.507 | 0.257 | 0.002 | –0.045 | 0.002 | 0.806 |
| 8 | 0.686 | 0.470 | 0.000 | 0.927 | 0.860 | 0.000 | –0.415 | 0.172 | 0.077 |

(for sites 2, 4, 5, and 8, an $r^2$ of 0.22, 0.09, 0.7, and 0.17, respectively).

The strong correlation with flow for both $NO_3^-$ and DOC at these sites indicates that similar processes or source waters control the concentration of both of these chemicals in the waters of Devil Canyon. Similarly, the negative correlation for $Ca^{2+}$ indicates that a different process is controlling the concentration of $Ca^{2+}$ in the Devil Canyon watershed. Possibly a flushing out process[9] of water and dissolved materials from soils may be responsible for the high $NO_3^-$ and DOC concentrations seen at higher flows. However, pore water flushing tends to produce a profound hysteresis (rising limb concentrations are usually higher than falling limb concentrations) in streamwater DOC and $NO_3^-$ concentrations, and at least at the larger sites (2, 5, and 8) we do not observe hysteresis. An alternative hypothesis for the higher $NO_3^-$ and DOC concentrations observed at higher flows may be due to increased hydraulic gradients in the subsurface, decreasing the residence time of waters in the subsurface and limiting the amount of time for denitrification to occur. Since both DOC and $NO_3^-$ are substrates in the microbial reactions of denitrification[11], increased residence time would decrease the concentrations of both $NO_3^-$ and DOC if denitrification were a major process controlling these two chemicals. This second hypothesis could explain the variability observed in the larger groundwater-dominated streams of Devil Canyon, since there are extensive riparian areas and much of the sampling is taken during baseflow conditions and not during active storm events.

The site groupings identified above can be further described by the physical processes that appear to be controlling $NO_3^-$ concentrations. Two sites appear to be dominated by deep groundwater, site 1 and site 7. Site 1 is a spring sampled at its source, so we know that it has little interaction with a riparian zone of any sort. Site 7 has relatively consistent and large flows and geochemically is enriched in $Ca^{2+}$ (concentrations of almost 100 mg $l^{-1}$ as opposed to average for the other sites of 60 mg $l^{-1}$), indicating that it has deep groundwater as its source. Sites 3 and 6 appear to be dominated by water from relatively shallow flowpaths (low $Ca^{2+}$ concentrations of ~20 mg $l^{-1}$), possibly more indicative of terrestrial processes than the groundwater-dominated streams like site 1 and site 7. Both have relatively low flows, with site 3 being an ephemeral stream, and site 6 having very low flows consistently throughout the year (and sometimes drying out, as it has this past summer). Sites 3 and 6 oftentimes show very low $NO_3^-$ concentrations, high DOC concentrations, and relatively low $Ca^{2+}$ concentrations, with the exception that at site 3, at the onset of streamflow, there is a very distinct flush of high $NO_3^-$ concentrations followed by low concentrations, as has been observed at many ephemeral streams in seasonally dry climates[6,12,13]. Site 3 also has a noticeable concurrent and similar flush of DOC (data not shown). These observations indicate that these two sites are most closely tied to the terrestrial biogeochemistry of their watersheds. The remaining sites (2, 4, 5, and 8) might be termed the perennial streams. These streams generally have a very strong correlation (positive or negative) between flow and $NO_3^-$, DOC, and $Ca^{2+}$ (correlations are weakest for site 4, with $Ca^{2+}$ being especially weak), unlike the ephemeral streams (sites 3 and sites 6). These streams are no doubt the most perennial of the streams we sampled, since their riparian areas are dominated by alder trees and they are deep groundwater-dominated streams for most of the sampling period. They also represent the streams most likely to be surveyed as indicators of ecosystem processes (whether terrestrial or aquatic) in southern California, since they actually flow throughout much of the year and would generally be accessible from the valley floor. It might be assumed that much of the $NO_3^-$ we observe in these streams originated as N fixed by the white alder that is commonly present in the riparian zones of these four streams. Alders are not a likely source since all other evidence points to the riparian areas being net N sinks (lower concentrations of $NO_3^-$ at site 2 and even lower ones at site 8, as compared to site 5). Additionally, we have conducted several longitudinal surveys down the stream, and the results of these surveys indicate consistently dropping $NO_3^-$ concentrations between site 2 and site 5 in all seasons (Fig. 5).

Overall, the causes of the variable $NO_3^-$ concentrations in streamwater among our sites in the Devil Canyon catchment are linked to the high rates of atmospheric deposition and persistent terrestrial ecosystem N limitation. Forests in the summer-dry climate of southern California, and other semiarid ecosystems with chronic N deposition, are especially prone to open (nonconservative) N cycling because of (1) active nitrification during all

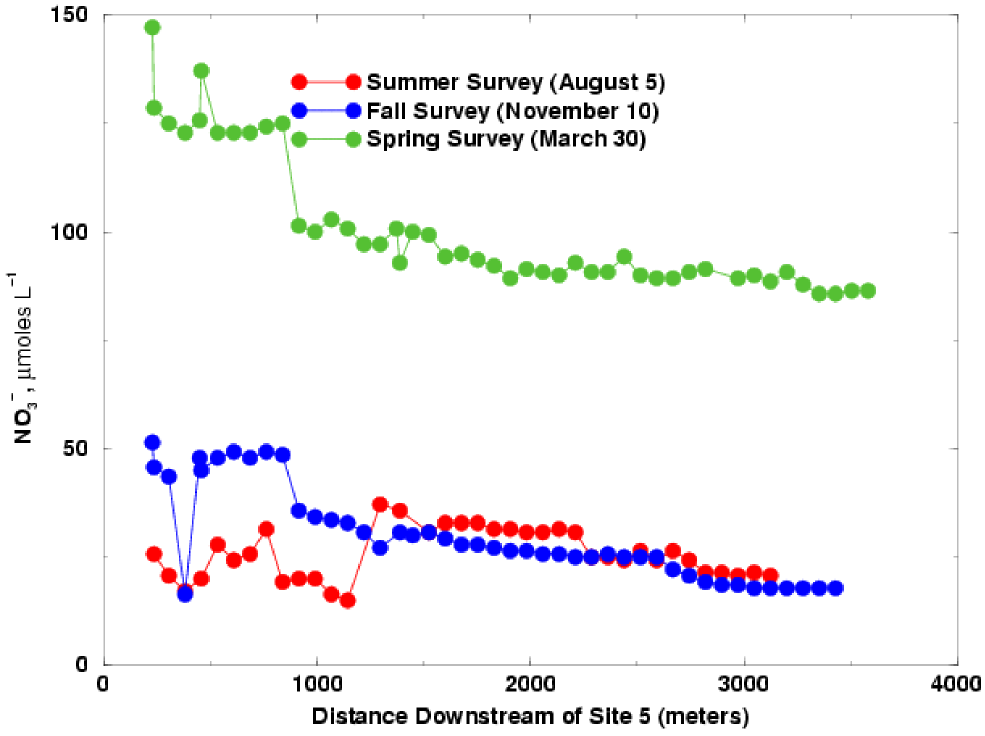

**FIGURE 5 :** Stream $NO_3^-$ concentrations against distance downstream from site 5. Note consistent decline in $NO_3^-$ with distance downstream.

stages of N saturation and (2) temporal asynchrony between hydrologic fluxes (winter) and plant N demand (spring/summer)[4]. Steep slopes and coarse-textured soils, typical in many watersheds (including all of these), also contribute to high $NO_3^-$ concentrations, especially during the wet winter season. Montane ecosystems with a Mediterranean climate are highly prone to boom or bust conditions in terms of hydrologic fluxes and associated effects of severe drying/wetting cycles. During extended dry periods, soil moisture is low and limits movement of soil water to deep percolation. The limited moisture also causes vegetation to shut down or limits its activity. Biological activity in the upper soil layers is also limited, and dry-deposited nitrogenous pollutants accumulate to very high levels in the vegetation canopy and in the upper soil layers[6,14,15]. The first precipitation events of the hydrologic year wash out N from the atmosphere and wash off N pollutants from vegetation, and transport concentrated pulses of N and other nutrients into the soil. This nutrient solution input into the soil stimulates microbial and root activity. Stimulated N mineralization and nitrification rates result in further pulses of released $NO_3^-$ into the soil solution[14,16]. Thus when vegetation begins growth further into the rainy season or when temperatures warm up in the spring, the soils are depleted of inorganic N and groundwaters have high levels of $NO_3^-$ that are observed in streams and springs throughout the growing season.

The flush of $NO_3^-$ at the beginning of flow at site 3 helps illustrate this linkage between the terrestrial systems and the high groundwater nitrate that we observe and bolsters our asynchrony hypothesis of biogeochemical control for Mediterranean climates. This initial flush of $NO_3^-$ is probably due to a washing out of the built up N deposition and soil mineralization as well as the nitrification of the previous dry season. This initial flush of $NO_3^-$ out of Mediterranean climate watersheds has been observed by others [J. Sickman, personal communication, 6,13], and this initial pulse may always exist even in the absence of atmospheric N deposition in seasonally dry ecosystems, as shown in several studies at the Jornada Long-Term Ecological Research Site[12,17]. The loss of $NO_3^-$ may be due to soil microbial and plant processes active at the seasonal transition in Mediterranean and other seasonally dry ecosystems, and may play a role in keeping these ecosystems N limited (J. Sickman and J. Schimel, personal communication). This regular pulse of $NO_3^-$ in seasonally dry ecosystems may be the reason for the relative resistance of the terrestrial portion of Mediterranean ecosystems to the effects of N deposition. Since they normally leach $NO_3^-$ at the beginning of the wet season, the added $NO_3^-$ from deposition may just be added to this pulse. Additionally, much of the $NO_3^-$ in the groundwater is leached into the groundwater during the early winter wet period when this high $NO_3^-$ is observed in soil water (M. Fenn, unpublished data) and ephemeral streamwater in Devil Canyon. This $NO_3^-$ leached into groundwater is then what is observed in the streams of Devil Canyon during baseflow conditions. Similar processes of high $NO_3^-$ soil waters being leached by wetter winter conditions into groundwaters have been observed by others in the Catskills Mountains of the northeastern U.S.[18] and at the Turkey Lakes watersheds in Ontario, Canada[10]. These similar results in two very different climates indicate that in systems where soil N conditions are hydrologically disconnected from streams for part of the year, high baseflow concentrations may not be indicative of N excess in terrestrial systems. In the Catskills and in Ontario this disconnect appears to happen in a limited set of catchments with specific geologic and topographic settings[10,18]. In Mediterranean climates the disconnect between terrestrial systems and aquatic ecosystems is probably a more normal condition; by definition streams that flow in late summer must be disconnected from much of the landscape since much of the landscape is in water stress[19].

The data indicate that the traditional analysis of N saturation in aquatic systems[3] may not apply to seasonally dry ecosystems. In the traditional analysis, the high baseflow concentrations of $NO_3^-$ that we observe at all of our sites other than 3 and 6 would be indicative of stage 2 of N saturation. However, the low baseflow concentrations at sites 3 and 6, the two sites tied most closely to terrestrial processes, indicate that the terrestrial ecosystems in Devil Canyon are still N limited. Additionally, fertilization experiments in the San Bernardino Mountains show that sites affected by N deposition still have a significant growth response when additional fertilizer is added to the soils, indicating that these forests are still N limited (M. Fenn, unpublished data). Perennial streams can have high baseflow/growing season nitrate levels due to earlier movement of nitrate from the surface and plant rooting zones to groundwaters; most of this movement takes place early in the rainy season when plant N demand is low.

## CONCLUSIONS

Preliminary evidence from the Devil Canyon watershed indicates that high baseflow $NO_3^-$ may not be indicative of N saturation of the surrounding terrestrial ecosystem but are instead indicative of hydrologic controls on ecosystem biogeochemistry in seasonally dry catchments. The high concentrations are evidence of a disconnect between the response of terrestrial and aquatic ecosystems to heightened levels of N deposition in seasonally dry ecosystems. While these processes appear to protect terrestrial ecosystems, they also appear to put aquatic ecosystems at greater risk of eutrophication from atmospheric N deposition in seasonally dry ecosystems. These results are preliminary and need to be confirmed by further study in these catchments as well as plot-based leaching studies and isotopic studies to ascertain the movement of $NO_3^-$ across and through the landscape.

## ACKNOWLEDGMENTS

This research was funded by the University of California Water Resources Center and by the University of California Agricultural Experiment Station. Pete Clark, Jeff McGovern, Megan Robinson, Bridgette Valeron, Jim Strong, and Peggy Resketo assisted in field sampling and laboratory analyses that contributed to this manuscript.

| | | |
|---|---|---|
| Received: | July | 27, 2001 |
| Revised: | October | 11, 2001 |
| Accepted: | October | 25, 2001 |
| Published: | November | 7, 2001 |
